# Five testicles in the genital area of a thirteen-month-old baby: a case report

**DOI:** 10.1186/s12894-020-00681-8

**Published:** 2020-08-20

**Authors:** Telma Zahirian Moghadam, Hamed Mohseni Rad, Hamed Zandian, Ali Hosseinkhani

**Affiliations:** 1 Social Determinants of Health Research Center, Ardabil University of Medical Sciences, Ardabil, Iran; 2 Department of Surgery, School of Medicine and Allied Medical Sciences, Imam Reza Hospital, Ardabil University of Medical Sciences, Ardabil, Iran

**Keywords:** Testicles, Genital area, Undescended testis (UDT)

## Abstract

**Background:**

Polyorchidism is a congenital anomaly of the urogenital system and means more than two testes. It is a rare phenomenon, where there are no more than 200 reported cases in the literature. In this case, we report a 13-month year’s old case with five testicles.

**Case presentation:**

We report a rare five testicles in the genital area of a 13-month-old baby. The initial diagnosis was undescended testis (UDT) based on ultrasound findings where a testis in the abdomen and a testis in the inguinal canal were detected. Surgery with general anaesthesia was performed to diagnose and treat this case. Before surgery, four HCG 1200u injections were administered. During the operation, it was determined that the case had had five testicles. Testicles were on the left in the proximal inguinal canal, and the sac hernia was ligated parallel to the inner ring. The patient was followed up several times after surgery by a urologist, and the results showed that there were no problems, and the intervention was uncomplicated.

**Conclusion:**

Based on the result, it is not possible to diagnose such cases only by examination or ultrasound in infant patients, as the patient often presents with undescended testis (UDT), so the disease is diagnosed only through surgery.

## Background

One of the congenital anomalies of the urogenital system is polyorchidism or supernumerary testis, which means more than two testes. It is a sporadic phenomenon, where there are no more reported cases in the literature. More than 50% of the all reported cases (about less than 200 cases) are detected in people between 10 to 25 years old [1, 2] and most of the discovered cases had less than four testicles [3]. In this case, we report a 13-month old case with five testicles.

## Case presentation

The case was a 13-month-old baby with 9.7 kg, which was 3.5 kg at birth, and second male child of a family habitant in a village in Ardabil, Iran. He was referred to the hospital because of undescended testis (UDT) with a testis in the abdomen and one inguinal canal that was detected by ultrasound. Because the case was infant, the family history was asked to examine the problem in more detail. Accordingly, there was UDT history in the family, where the baby’s old brother also underwent orchiopexy surgery 4 years ago for UDT. During the pregnancy, the progesterone was injected to the mother for Hemorrhage every day for a week. The pregnancy was healthy and the baby is born by cesarean section.

Because of complete similar appearance to testis as the images show, we did not perform biopsy fearing of primary testicular delicate perfusion impairment. The baby referred to our centre because of bilateral UDT and nonpalpable testicle on the right side. However, sonography revealed both testicles in the proximal inguinal canal. The scrotum was not grown, HCG therapy had been started by pediatrician, and Other clinical signs were typical.

**Fig. 1:**
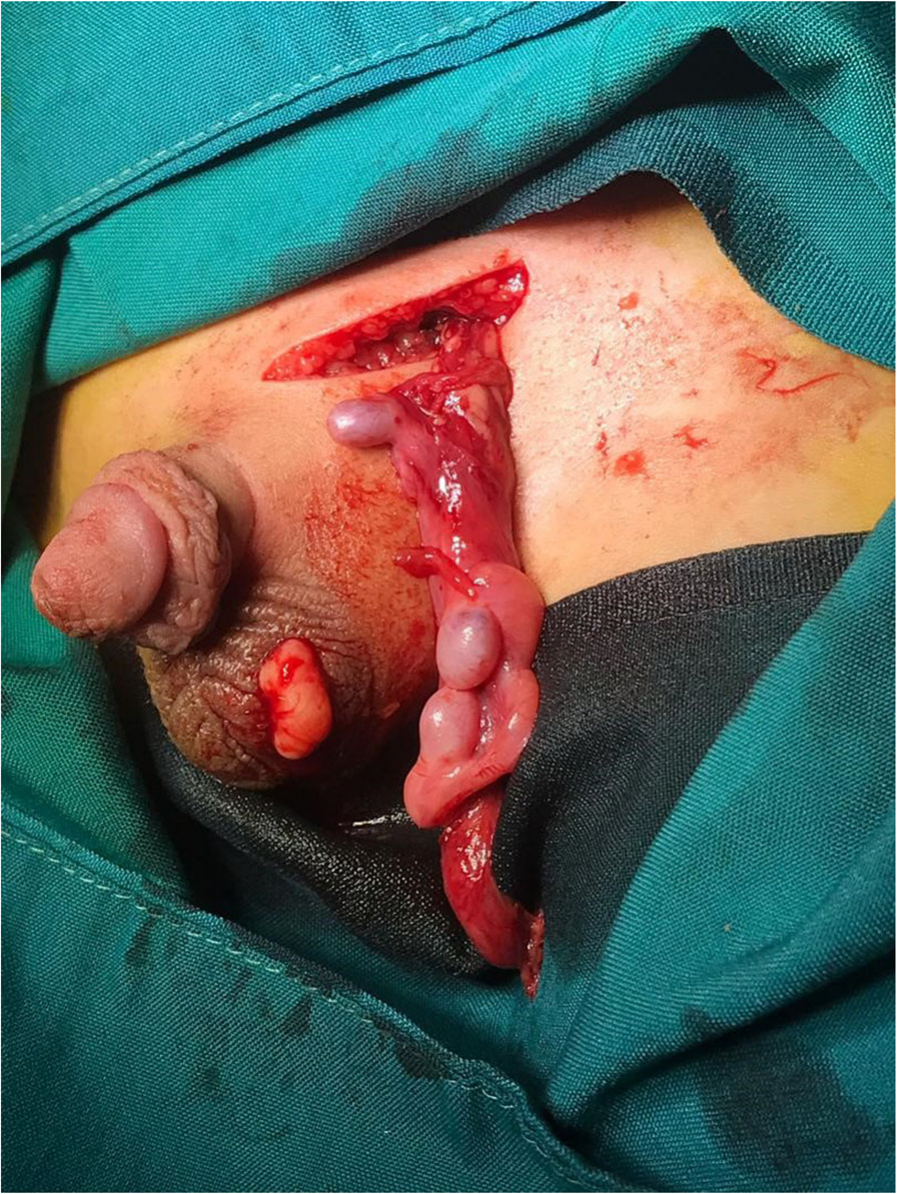
left testis, where the testicles were on the left in the proximal inguinal canal, and the sac hernia was ligated parallel to the inner ring

Surgical intervention was considered for the patient. Before surgery, four HCG 1200u injections were administered to the patient in four times at 1 month (one for per week) for testicular descent. However, the intervention did not completely resolve the problem, and there were no more diagnostic challenges. The patient underwent surgical procedure with general anaesthesia to diagnosis and as a final treatment stage. It was diagnosed that the testicles were on the left in the proximal inguinal canal and the sac hernia was ligated parallel to the inner ring. The left testis was a small component in the proximal without the epididymis. Two testes were closely connected in the lower part by a common epididymis, which all of them were situated in the left scrotum (Fig. 1).

Also, two testes in the proximal inguinal canal were connected by a long epididymis with the incision in the right inguinal region. We did not remove supernumerary testis, which lacks vas or epididymis, because of afraid of impairing to main testis delicate artery.

**Fig. 2:**
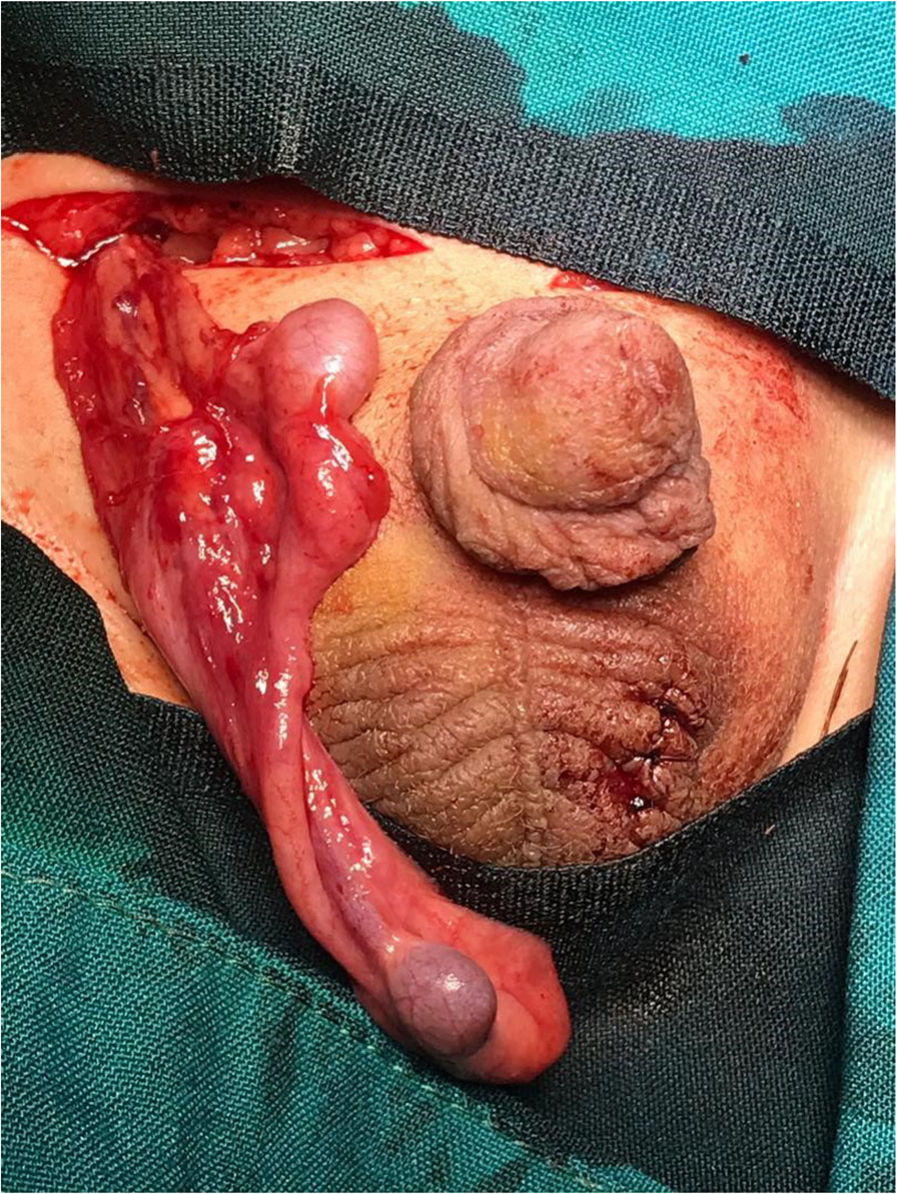
right testis, where a long epididymis connected two testes in the proximal inguinal canal with an incision in the right inguinal region

However, we do not perform genetic evaluations because of cost lacking. We played ultra-sonography before surgery. The patient was followed and examined by a urologist for placement of testis in the scrotum sac within 48 h, 10 days, one and 3 months after the surgery. We accomplished scrotal sonography after surgery and detected all five testicles with two main testicles in standard size rendering to age and also supernumerary ones in smaller sizes. Finally, the 12 months later, sonography results in the testicles showed that there were no problems, and the intervention was uncomplicated according to the baby age (Fig. 2).

## Discussion and conclusion

Blasisus in 1670 was the first one who described polyorchidism in an autopsy material, incidentally. Ahlfeld made the first histological description in 1880, and the first clinical case was reported on by Lane in 1895 [2, 4, 5]. The aetiology of polyorchidism is related to embryological period; however, this claim is not entirely clear [5, 6].

Leung classified polyorchidism into four groups based on testis embryology: 1) polyorchidism with novas differences or epididymis in the supernumerary testis, 2) testes sharing these two structures with the ipsilateral testicle, 3) testes with their epididymis and sharing the vas deferens, and 4) supernumerary testes with their annexes [7]. The patient was considered to be in group 1.

The scrotum is the most common region that Extra testicles could be seen. The other testis is usually seen on the left side [4], similar to our study. Three and four testes are the most common form [4, 7]. Five testes have not been reported so far, and our case is the first.

The aetiology is still unclear. It is thought to be an accidental division of the genital ridge before 8th week of gestation. Although there are many embryological theories in the literature, none are sufficient to explain the pathogenesis of polyorchidism alone [7]. Our report showed that there were two right testicles with one common epididymis and one left testis without epididymis. The most common disorders of the male genital tract, which are diagnosed by clinical examination and sonography, are undescended testis, hydrocele, hypospadias, inguinal hernia, micropenis or penis enlargement and testicular defect. Treatment is often done through surgery [8, 9]. The presence of five testes, in this case, was one of the rare disorders of the genital tract in children. Aetiology and mechanism of polyorchidism are not entirely defined. However, some genetic and gestational factors may be involved, because the baby’s brother also underwent orchiopexy surgery 4 years ago for UDT and his mother was injected progesterone every day for a week of pregnancy for Hemorrhage. Therefore, further studies are recommended to investigate the factors affecting these disorders.

The case with five testes is rare and undesirable in children. In both sides, testicles were in the proximal inguinal canal and nor in the abdomen. Diffusion-weighted imaging (DWI) combined with conventional magnetic resonance imaging (MRI) increase sensitivity and specificity of MRI for nonpalpable UDT detection [10]. While in our case, because of clinical finding and sonography confirmation, we did not render an abdominal MRI. Laparoscopy is standard in abdominal nonpalpable testicles, but it could be omitted if sonography confirmed their inguinal presence [11]. It has been shown that along with surgery, neo-adjuvant human chorionic gonadotropin (HCG) therapy may induce and improve testis descending [12]. In this case, surgery was performed to diagnose and treat this case. The patient needs to be monitored at different intervals. Rare cases should be handled carefully; particularly in patients with reported undescended testicles by ultrasound. During surgery, it is essential to note that more testicles may be found and we must search carefully. It is also advisable during orchiopexy, and all testes are mixed without separation to prevent damage to the epididymal system, sperm transfer, and the spermatozoa.

## Data Availability

Not applicable.

## Abbreviations

UDT: Undescended testis
